# Attitudes and consumption habits of energy drinks among adolescents and young adults in a Spanish population

**DOI:** 10.3934/publichealth.2025002

**Published:** 2025-01-03

**Authors:** Eduardo Sánchez-Sánchez, Nuria Trujillo-Garrido, Jara Díaz-Jimenez, Alejandro García-García, Miguel A Rosety, Manuel Bandez, Manuel Rosety-Rodriguez, Francisco J Ordonez, Ignacio Rosety, Antonio J Diaz

**Affiliations:** 1 Internal Medicine Department, Punta de Europa Hospital, 11207, Algeciras, Cádiz, Spain; 2 Nursing and Physiotherapy Department, School of Nursing, University of Cádiz, C/Venus s/n, 11207 Cádiz, Spain; 3 Biomedical Research and Innovation Institute of Cadiz (INIBICA), Puerta del Mar. University Hospital, University of Cadiz, 11009 Cadiz, Spain; 4 Doctoral School of the University of Cádiz (EDUCA), Campus Cádiz, Edificio Hospital Real (Primera Planta), Plaza Falla 8, 11003 Cádiz Spain; 5 Sports Sciences Department, School of Education Sciences, University of Cádiz, Avda. Rep. Saharahui 11519 Puerto Real, Spain; 6 Biomedicine Department, School of Medicine, University of Cádiz, Plaza Fragela s/n 11003 Cadiz Spain; 7 Medicine Department, School of Medicine, University of Cádiz, Plaza Fragela s/n 11003 Cadiz Spain; 8 Human Anatomy, School of Medicine, University of Cádiz, Plaza Fragela s/n 11003 Cadiz Spain

**Keywords:** energy drink, alcohol consumption, adverse events, adolescent, young adult

## Abstract

Nowadays, the consumption of energy drinks (ED) is increasing exponentially in Western society. This has been widely associated with physical (arrhythmias, headaches, etc.), psychological (anxiety, depression, etc.), and social issues (risky behaviors such as excessive alcohol consumption, etc.). The present study aimed to investigate the consumption habits of energy drinks (ED) among adolescents and young adults in the Spanish population and their attitudes toward these drinks, as well as the factors influencing their consumption. A cross-sectional descriptive study based on a voluntary online questionnaire was conducted with a sample of 387 participants. Amongst participants, 38.8% consumed ED, and the youngest (14–18 years old) in this group were the most likely to mix them with alcohol and the least likely to consider them harmful (32.1%, *p* < 0.001; and 8.9%, *p* < 0.002, respectively). Male respondents and people who vaped were more likely to consume ED (*OR* = 2.94, *CI* = 1.76–4.93, *p* < 0.001; and *OR* = 3.18, *CI* = 1.91–8.00, *p* < 0.001, respectively). Social and healthcare policies should be proposed in order to reduce the consumption of ED, particularly among young people, provided that it is associated with other risky behaviors and the occurrence of adverse events.

## 1. Introduction

Energy drinks (EDs) are beverages that are geared toward enhancing mental and physical stimulation. In fact, they contain energy-boosting ingredients, such as caffeine, taurine, herbal extracts, sugar, and B vitamins [1]. However, their standardization and the increasing variety of EDs on the market have changed the trend. EDs are the fastest-growing segment in the beverage industry, only surpassed by bottled water. According to Aonso-Diego et al. [2], the worldwide pooled prevalence of ED use in 2022 was estimated to be 32.3% (95% *CI * =  28.8–35.8; *I^2^* =  99.82) in the past 30 days, 21.6% (95% *CI*  =  18.7–24.5; *I^2^* =  99.95) in the past 7 days, and 8.82% (95% *CI*  =  6.3–11.4; *I^2^*  =  99.95) daily ED use. Regarding their consumption among the pediatric and adolescent population, a recent study revealed that more than half of children worldwide consume energy drinks weekly or monthly [3]; according to a study conducted in Spain, 30.9% of adolescents consumed EDs in the last week [4]. Furthermore, ED brands mostly advertise on social media by aligning their products with the common socio-cultural values and practices that are considered relevant by today's youth, such as masculinity, femininity, friendship, and leisure; thus, these brands show that their products have a regular place in their daily lives [5]. Several studies have shown the high prevalence of ED marketing on social media and live-streaming platforms, such as Twitch, Facebook, and YouTube games [5]–[8]. Specifically in Spain, it has been observed that 78.4% of Twitch users have identified advertising for two brands of EDs during the streams of two well-known Spanish streamers [9].

Several medical associations have warned about the risks posed by energy drinks to the younger population [4],[10]–[12], as the consumption of these beverages is emerging as a public health issue due to several factors. On one hand, their caffeine content often exceeds acceptable concentrations according to food regulations [13]; on the other hand, high levels of potentially toxic elements have been found in energy drinks, which can lead to acute or long-term intoxications [14]. Additionally, their use is associated with adverse physical and psychological effects, such as cardiovascular problems, headaches, stomach discomfort, anxiety, hyperactivity, and insomnia [4],[10],[12]. Sugar intake can also lead to overweight and obesity and is associated with an increased risk of type 2 diabetes [5]. Furthermore, energy drink consumption is also associated with risky behaviors [15] such as smoking or engaging in risky sexual activities [16]. Among these risky behaviors, alcohol consumption and vaping stand out [17]–[21].

Regarding alcohol, its consumption mixed with energy drinks is a particularly dangerous combination [22],[23]; the combination of both beverages increases the likelihood of experiencing the adverse effects derived from the stimulant characteristics of energy drinks while decreasing the sedative effects of alcohol consumption. This could lead to consuming more alcohol and even driving under its influence, partly because energy drinks counteract the effects of alcohol on driving skills [24]. On the other hand, the consumption of energy drinks is related to the use of electronic cigarettes [21],[25]. In fact, a study conducted in Helsinki with adolescents aged 12 to 13 found that one of the strongest predictors for vaping in both genders was the consumption of energy drinks [26].

Given all of this, authors such as Kaur et al. [27] argued that there is an immediate requirement for age guidelines, clear ingredient transparency, openly disclosing potential side effects, and, most importantly, educating consumers. However, there are still only a few studies on adolescent and young adult populations in Spain.

Therefore, the aim of our study is to examine the consumption habits of ED among adolescents and young adults in the Spanish population, their attitudes and beliefs toward such consumption, and the variables that could influence them.

## 2. Materials and methods

### 2.1. Study design and participants

A cross-sectional observational study was conducted in a population aged 14 to 34 years. A convenience snowball sampling method was used, where study participants spread the questionnaire to other participants without restrictions. The study population consisted of 387 participants.

### 2.2. Data collection instrument

No validated questionnaire was found for assessing ED consumption in the adolescent and/or young adult population. The authors developed a structured questionnaire based on existing questionnaires that evaluated tobacco and drug use [28],[29]. To ensure the clarity and relevance of the questions, a focus group of five people was consulted, which allowed some questions to be modified and ambiguities of content to be avoided. The focus group consisted of five individuals who met the inclusion and exclusion criteria. Only expressions that could hinder the understanding of the questionnaire were modified; however, the content of the questions was not altered.

The questionnaire was divided into three sections. The first section collected data on sociodemographic variables, such as age, gender, employment status, etc. The second section asked questions related to behavioral and lifestyle variables. The third section consisted of variables on ED consumption. The questionnaire questions can be seen in the different tables in the Results section.

### 2.3. Variables

BMI: The categories are divided into four categories: underweight for a BMI < 18.5 kg/m^2^, normal weight for a BMI from 18.5 to 24.9 kg/m^2^, overweight for a BMI from 25 to 29.9 kg/m^2^, obesity class I for a BMI from 3 to 34.9 kg/m^2^, obesity class II for a BMI from 35 to 39.9 kg/m^2^, and obesity class III for a BMI greater than or equal to 40 kg/m^2^ [30].

Educational level: To assess the knowledge and cultural level of the studied population, educational level was classified into five categories: below primary education level, primary education, secondary education (high school), tertiary education (undergraduate level), and graduate level (Master's degree or Doctoral degree).

Income level: Calculated based on the average Spanish income, which, according to data from the National Institute of Statistics in 2020 [31], was 18,490 €, and subsequently classified into three categories: high, medium, and low. The upper class encompassed participants whose income was 150% above the Spanish average income (more than 46,225 € annually); the middle class ranged from a minimum of 30% below the average income to a maximum of 150% above it (from 12,943 to 46,225 € annually); and the lower class corresponded to those whose income was below 30% of the average income (below 12,943 € annually). In the case of non-emancipated participants, the income level of their family group was taken into account.

### 2.4. Data collection

The questionnaire was administered online through social networks or content dissemination platforms by sending a link to the Google Forms free questionnaire platform [32]. Data collection was carried out in March and April 2020.

### 2.5. Statistical analysis

The statistical analysis utilized the SPSS 25 software. Initially, a descriptive analysis was conducted to assess the distribution of variables in the sample. Qualitative variables were depicted using frequency and percentage, while quantitative variables were presented through mean and standard deviation or dispersion. Following this, the Chi-squared test was employed to examine significant differences in the correlation between age and ED consumption. Additionally, correlations between vaping and ED consumption, as well as associations between vaping and independent variables, were investigated. A linear regression model was then applied, incorporating independent variables such as age, gender, place of residence, and employment status, with ED consumption as the dependent variable. Odds ratio (*OR*) values were derived, and statistical significance was determined with a *p*-value < 0.05.

### 2.6. Ethical considerations

This work was carried out following the Declaration of Helsinki (Fortaleza 2013). Data provided were anonymous, as no identifying or pseudonymized data were recorded, and no additional information was collected that could be attributed to the participant. Participation in the study was voluntary and no financial compensation was received. Participants were provided with an informed consent form, which had to be accepted before starting the questionnaire. This consent form stated that participants could withdraw at any time and that their responses would only be used to investigate the study's objectives.

## 3. Results

The questionnaire was answered by a total of 387 respondents, among which 72.1% were women (*n* = 279) and 27.9% (*n* = 108) were men. The participants were mostly between 19 and 22 years (43.7%, *n* = 169). Regarding the place of residence, 78.8% lived with family at home, while 19.1% and 2.1% lived in shared housing and alone, respectively. Of the participants, 81.7% belonged to the medium socioeconomic class, and 64.3% were students (at the time of the survey). Of the students, 54% were studying at a university (Table 1).

**Table 1.** General characteristics of the participants.

**Project**	***n***	**%**
**Gender**		
• Male	108	27.9
• Female	279	72.1
**Age range**		
• 14–18 years	56	14.5
• 19–22 years	169	43.7
• 23–26 years	126	32.5
• 27–30 years	21	5.4
• 31–34 years	15	3.9
**Residence**		
• Familiar	305	78.8
• Shared accommodation	74	19.1
• Alone	8	2.1
**Education level**		
• Primary education	13	3.3
• Secondary education	129	33.3
• Tertiary education	209	54.0
• Graduate level	35	9.0
• Below primary education level	1	0.3
**Income level**		
• Low-income level	63	16.2
• Medium-income level	317	81.7
• High-income level	8	2.1
**Currently, what do you do?**		
• Studying	249	64.3
• Working	70	18.1
• Studying and working	68	17.6

Note: Descriptive analysis of the sample characteristics studied.

Among the participants, 61.2% reported not consuming ED, while 38.8% reported ED consumption. No statistically significant differences were found in the different age groups.

The most common frequency of consumption among participants in all age groups was 1–3 times per week (33.7%) (excluding the 61.2% who reported never drinking EDs). Of the participants aged 27–30 years and 14–18 years, 9.5% and 8.9%, respectively, reported consuming ED once or twice a day, with no statistically significant differences between age groups (Table 2).

**Table 2.** Responses to the questionnaire questions on attitudes and consumption habits of energy drinks.

**Questions**	**Age range (*n*, %)**					**Total**	***p***
	**14–18**	**19–22**	**23–26**	**27–30**	**31–34**		
**Are you a regular consumer of energy drinks?**							
• Yes	28 (50.0)	63 (37.3)	50 (39.7)	7 (33.3)	2 (13.3)	150 (38.8)	0.110
• No	28 (50.0)	106 (62.7)	76 (60.3)	14 (66.7)	13 (86.7)	237 (61.2)	
**How often do you consume energy drinks?**							
• 5 times a day	0 (0.0)	1 (0.6)	0 (0.0)	0 (0.0)	0 (0.0)	1 (0.3)	0.291
• 3–5 times a day	1 (1.8)	0 (0.0)	1 (0.8)	0 (0.0)	0 (0.0)	2 (0.5)	
• 1–2 times a day	5 (8.9)	2 (1.2)	3 (2.4)	2 (9.5)	0 (0.0)	12 (3.1)	
• 4–6 times a week	1 (1.8)	3 (1.8)	1 (0.8)	0 (0.0)	0 (0.0)	5 (1.3)	
• 1–3 times a week	21 (37.5)	55 (32.5)	45 (35.7)	5 (23.8)	2 (13.3)	130 (33.7)	
• Never	28 (50.0)	108 (63.9)	75 (60.3)	14 (66.7)	13 (86.7)	237 (61.2)	
**Do you think that consumption of energy drinks is harmful?**							
• Yes	43 (76.8)	162 (95.9)	116 (92.1)	20 (95.2)	15 (100.0)	356 (92.0)	0.002
• No	5 (8.9)	2 (1.2)	4 (3.2)	1 (4.8)	0 (0.0)	12 (3.1)	
• Do not know	8 (14.3)	5 (3.0)	6 (4.8)	0 (0.0)	0 (0.0)	19 (4.9)	
**Do you think that energy drinks are healthier than soft and/or sugary drinks like Cola or Fanta?**							
• Yes	4 (7.1)	2 (1.2)	6 (4.8)	0 (0.0)	0 (0.0)	12 (3.1)	0.343
• No	47 (83.9)	158 (93.5)	114 (90.5)	20 (95.2)	14 (93.3)	353 (91.2)	
• Do not know	5 (8.9)	9 (5.3)	6 (4.8)	1 (4.8)	1 (6.7)	22 (5.7)	
**Do you mix energy drinks with alcohol?**							
• Yes	18 (32.1)	26 (15.4)	12 (9.5)	4 (19.0)	0 (0.0)	60 (15.5)	<0.001
• No	15 (26.8)	43 (25.4)	42 (33.3)	4 (19.0)	2 (13.3)	107 (27.6)	
• Not applicable*	23 (41.1)	100 (59.2)	72 (57.1)	13 (61.9)	13 (86.7)	220 (56.8)	
**Why do you use energy drinks?**							
• Study	9 (16.1)	38 (12.5)	16 (12.7)	1 (4.8)	0 (0.0)	64 (16.5)	<0.001
• Pleasure	22 (39.3)	28 (16.6)	25 (19.8)	4 (19.0)	0 (0.0)	79 (20.4)	
• Being active	4 (7.1)	6 (3.6)	14 (11.1)	2 (9.5)	2 (13.3)	28 (7.2)	
• Not applicable*	23 (41.1)	100 (59.2)	72 (57.1)	13 (61.9)	13 (86.7)	216 (55.8)	

Note: * participants who do not consume ED. Chi-squared test. Statistically significant values: *p* ≤ 0.05.

Among all participants, 92.0% considered ED consumption harmful to health, but only 8.9% of the youngest participants (14–18 years) acknowledged this. There were statistically significant differences found in each age group. Additionally, 91.2% of the participants reported that these beverages were not healthier than other soft drinks. Among the participants who consumed ED, 15.5% mixed them with alcohol and 27.6% consumed them as is. When looking at the values by age group, statistically significant differences are found. For example, participants aged 14–18 years reported mixing alcohol with ED more frequently than just single ED consumption (32.1% *vs*. 26.8%, respectively). However, in the other age groups, this correlation was reversed, and the most frequent response was that they consumed ED without alcohol (Table 2).

The most common reason for ED consumption was pleasure (20.4%), followed by “in order to study” (16.5%), and statistically significant differences were found in the different age groups (*p* < 0.001). The age group comprising 14–18-year-olds consumed ED for pleasure most frequently (39.3%) (Table 2).

We assessed whether ED consumption was related to different physical or behavioral variables, such as BMI, self-perceived health condition, physical activity, quality of sleep and rest, nightlife, work or academic performance, and vaping habits. We found that those who consumed ED more often were more likely to report difficulties in their work and/or academic performance than those who did not (14.7% *vs*. 5.5%; *p* = 0.004). 13.2% of the participants vaped, and this habit was more prevalent among participants who consumed ED (23.3% *vs*. 6.8%; *p* < 0.001) (Table 3).

Additionally, a linear regression model was applied with ED consumption as the dependent variable (Table 4). The results revealed that male participants had 2.94 times higher odds of consuming ED than the female participants (*CI* = 1.76–4.93; *p* < 0.001). However, respondents aged between 31 and 34 years and postgraduates showed 0.17- and 0.22-times lower odds of consuming ED, respectively (*OR* = 0.17, *CI* = 0.01–0.89, *p* = 0.054; and *OR* = 0.22, *CI* = 0.04–1.04, *p* = 0.0062, respectively). Finally, participants who vaped frequently had an odds ratio of 3.84 for ED consumption (*CI* = 1.91–8.00; *p* < 0.001) (Table 4).

Regarding the correlation between frequency of consumption and the other variables, we observed that, among the participants who reported never drinking ED, 81.2% were male and 18.8% were female (*p* < 0.001). Also, 89.8% of the participants who reported an ED consumption frequency of 1–3 times a week acknowledged that it was harmful to their health (*p* = 0.002) (Table 5).

**Table 3.** Relationship between variables and energy drinks.

**Project**	**Energy drinks**		**Total**	***p***
	**YES^a^**	**NO^b^**		
**Do you vape?**				
• Yes	35 (23.3)	16 (6.8)	51 (13.2)	<0.001
• No	115 (76.7)	221 (93.2)	336 (86.8)	
**BMI**				
• Underweight	7 (4.7)	17 (7.2)	24 (6.2)	0.061
• Normal weight	83 (55.3)	159 (67.1)	242 (62.5)	
• Overweight grade I	25 (16.7)	29 (12.2)	54 (13.9)	
• Overweight grade II	23 (15.3)	21 (8.9)	44 (11.4)	
• Type I obesity	8 (5.3)	10 (4.2)	18 (4.6)	
• Type II obesity	2 (1.3)	1 (0.4)	3 (0.8)	
• Type III obesity	2 (1.3)	0 (0.0)	2 (0.5)	
**How do you think is your health condition in comparison to others?**				
• Very good	25 (16.7)	48 (20.3)	73 (18.8)	0.304
• Good	90 (60.0)	152 (64.1)	242 (62.5)	
• Regular	32 (21.3)	33 (13.9)	65 (16.8)	
• Bad	3 (2.0)	3 (1.3)	6 (1.5)	
• Very bad	0 (0.0)	1 (0.4)	1 (0.3)	
**How many hours do you sleep during nighttime?**				
• <4 h	1 (0.7)	1 (0.4)	2 (0.5)	0.152
• 4–6 h	22 (14.7)	23 (9.7)	45 (11.6)	
• 6–8 h	104 (69.3)	157 (66.2)	261 (67.4)	
• >8 h	23 (15.3)	56 (23.6)	79 (20.4)	
**Do you rest well?**				
• Yes	102 (75.5)	179 (75.5)	281 (72.6)	0.268
• A bit	41 (21.1)	50 (21.1)	91 (23.5)	
• No	7 (3.4)	8 (3.4)	15 (3.9)	
**Do you practice physical exercise?**				
• Daily	8 (5.3)	17 (7.2)	25 (6.5)	0.791
• 5/week	26 (17.3)	38 (16.0)	64 (16.5)	
• 3/week	53 (35.3)	79 (33.3)	132 (34.1)	
• 1/week	30 (20.0)	41 (17.3)	71 (18.3)	
• Never	33 (22.0)	62 (26.2)	95 (24.5)	
**How is the intensity of the exercise (measured with speech test)?**				
• Light	34 (22.7)	48 (20.3)	82 (21.2)	0.889
• Moderate	73 (48.7)	115 (48.5)	188 (48.6)	
• Intense	12 (8.0)	18 (7.6)	30 (7.7)	
• Not applicable	31 (20.7)	56 (23.6)	87 (22.5)	
**How often do you go out on nightlife?**				
• >2/week	26 (17.3)	18 (7.6)	44 (11.4)	0.066
• 2/week	31 (20.7)	52 (21.9)	83 (21.4)	
• 1/week	31 (20.7)	51 (21.5)	82 (21.2)	
• 1–3/month	40 (26.7)	64 (27.0)	104 (26.9)	
• <1/month	16 (10.7)	39 (16.5)	55 (14.2)	
• Never	6 (4.0)	13 (5.5)	19 (4.9)	
**Academic/job performance:**				
• Very good	38 (25.3)	89 (37.6)	127 (32.8)	0.004
• Good	88 (58.7)	131 (55.3)	219 (56.6)	
• With difficulties	22 (14.7)	13 (5.5)	35 (9.0)	
• Do not know	2 (1.3)	4 (1.7)	6 (1.5)	
**Do you have concentration difficulties?**				
• Yes	21 (14.0)	20 (8.4)	41 (10.6)	<0.001*
• Sometimes	87 (58.0)	147 (62.0)	234 (60.5)	
• No	42 (28.0)	70 (29.5)	112 (28.9)	

Note: BMI, body mass index; a: participants who consume ED; b: participants who do not consume ED; Chi-squared test. Statistically significant values: *p* ≤ 0.05.

**Table 4.** Odd ratios for energy drink consumption in multivariate regression.

**Variables**	**Energy drinks consumption**		
	***OR***	**95% *CI***	***p***
**Gender (female)**			
• Male	2.94	1.76–4.93	<0.001
**Age range (years) (14–18)**			
• 19–22	0.85	0.40–1.81	0.676
• 23–26	0.73	0.32–1.66	0.459
• 27–30	0.66	0.16–2.49	0.551
• 31–34	0.17	0.01–0.89	0.054
**BMI (underweight)**			
• Normal weight	1.35	0.52–3.82	0.551
• Overweight grade I	1.72	0.58–5.50	0.336
• Overweight grade II	2.50	0.81–8.27	0.118
• Type I obesity	1.68	0.42–6.93	0.460
• Type II obesity	0.00	0.00–NA	0.987
• Type III obesity	0.00	0.00–NA	0.987
**Residence (familiar)**			
• Shared accommodation	1.29	0.71–2.32	0.397
• Alone	3.55	0.73–20.3	0.123
**Currently, what do you do? (studying)**			
• Studying and working	1.01	0.52–1.92	0.975
• Working	1.17	0.57–2.40	0.655
**Socioeconomic level (high)**			
• Low	2.61	0.35–53.79	0.410
• Medium	2.61	0.38–52.01	0.394
**Study level (primary education)**			
• No formal education	0.00	NA–0.00	0.985
• Secondary education	0.83	0.21–2.98	0.780
• University degree	0.49	0.12–1.85	0.303
• Postgraduate degree	0.22	0.04–1.04	0.006
**Vape consumption (no)**			
• Yes	3.84	1.91–8.00	< 0.001

Note: Multivariate regression. Statistically significant values: *p* ≤ 0.05.

**Table 5.** Relation between variables and frequency of consumption.

**Project**	**How often do you consume energy drinks?**						***p***
	**>5/day**	**3–5 /day**	**1–2/day**	**4–6/week**	**1–3/week**	**Never**	
**Gender**							<0.001
• Male	1 (100.0)	1 (50.0)	4 (33.3)	1 (50.0)	76 (59.4)	194 (81.2)	
• Female	0 (0.0)	1 (50.0)	8 (66.7)	1 (50.0)	52 (40.6)	45 (18.8)	
**Age range**							0.291
• 14–18 years	0 (0.0)	1 (50.0)	5 (41.7)	1 (20.0)	21 (16.4)	28 (11.7)	
• 19–22 years	1 (100.0)	0 (0.0)	2 (16.7)	3 (60.0)	55 (43.0)	108 (45.2)	
• 23–26 years	0 (0.0)	1 (50.0)	3 (25.0)	1 (20.0)	45 (35.2)	76 (31.8)	
• 27–30 years	0 (0.0)	0 (0.0)	2 (16.7)	0 (0.0)	5 (3.9)	14 (5.9)	
• 31–34 years	0 (0.0)	0 (0.0)	0 (0.0)	0 (0.0)	2 (1.6)	13 (5.4)	
**Academic/job performance**							0.047
• Very good	0 (0.0)	1 (50.0)	3 (25.0)	1 (20.0)	32 (25.0)	0 (0.0)	
• Good	1 (100.0)	0 (0.0)	7 (58.3)	4 (80.0)	78 (58.6)	1 (100.0)	
• With difficulties	0 (0.0)	1 (50.0)	1 (8.3)	0 (0.0)	20 (15.6)	0 (0.0)	
• Do not know	0 (0.0)	0 (0.0)	1 (8.3)	0 (0.0)	1 (0.8)	0 (0.0)	
**Do you think that consumption of energy drinks is harmful?**							0.002
• Yes	1 (100.0)	2 (100.0)	7 (58.3)	5 (100.0)	115 (89.8)	1 (100.0)	
• No	0 (0.0)	0 (0.0)	3 (25.0)	0 (0.0)	4 (3.1)	0 (0.0)	
• Do not know	0 (0.0)	0 (0.0)	2 (16.7)	0 (0.0)	9 (7.0)	0 (0.0)	
**Do you have concentration difficulties?**							0.825
• Yes	0 (0.0)	0 (0.0)	2 (16.7)	0 (0.0)	18 (14.1)	0 (0.0)	
• Sometimes	1 (100.0)	2 (100.0)	6 (50.0)	3 (60.0)	74 (57.8)	1 (100.0)	
• No	0 (0.0)	0 (0.0)	4 (33.3)	2 (40.0)	36 (28.1)	0 (0.0)	
**How many hours do you sleep during nighttime?**							0.211
• <4 h	0 (0.0)	0 (0.0)	0 (0.0)	0 (0.0)	1 (0.8)	0 (0.0)	
• 4–6 h	0 (0.0)	1 (50.0)	2 (16.7)	1 (20.0)	17 (13.3)	0 (0.0)	
• 6–8 h	0 (0.0)	1 (50.0)	8 (66.7)	1 (20.0)	93 (72.7)	0 (0.0)	
• >8 h	1 (100.0)	0 (0.0)	2 (16.7)	3 (60.0)	17 (13.3)	1 (100.0)	
**Do you rest well?**							0.006
• Yes	1 (100.0)	0 (0.0)	7 (58.3)	3 (60.0)	89 (69.5)	1 (100.0)	
• A bit	0 (0.0)	1 (50.0)	4 (33.3)	2 (40.0)	34 (26.6)	0 (0.0)	
• No	0 (0.0)	1 (50.0)	1 (8.3)	0 (0.0)	5 (3.9)	0 (0.0)	

Note: Chi-squared test. Statistically significant values: *p* ≤ 0.05.

## 4. Discussion

The aim of the study is to examine the consumption habits of ED among adolescents and young adults in the Spanish population, as well as their attitudes and beliefs toward such consumption, and the variables that could influence them. Our study results show that 38.8% of the total sample reported ED consumption. However, it is especially noteworthy that 50% of the youngest participants (aged 14–18 years) reported ED consumption, although the results were not statistically significant. The results referring to the opinion of participants regarding whether Eds are harmful to health were statistically significant, with the group comprising participants aged 14–18 years being least likely to acknowledge any harmful effects (14–18 years: 8.9%; 19–22 years: 1.2%; 23–26 years: 3.2%; 27–30 years: 4.8%; and 31–34 years: 0%; *p* = 0.002). These results align with what has been observed in other studies showing that adolescents have less knowledge about the possible consequences of consuming ED and are, therefore, more likely to consume them [33]–[35].

In fact, if we consider knowledge level and experience, we observed that in our sample, older participants (aged 31–34 years) and postgraduates were less likely to consume Eds (*OR* = 0.17, *CI* = 0.01–0.89, *p* = 0.054; and *OR* = 0.22, *CI* = 0.04–1.04, *p* = 0.0062, respectively).

Regarding the reasons for ED consumption, 20.4% of the participants who consumed EDs cited the reason to be for, with younger people being more likely to cite this reason (14–18 years: 39.3%; 19–22 years: 16.6%; 23–26 years: 19.8%; and 31–34 years: 0%; *p* < 0.001). These data align with the findings of other studies, which reveal that one of the most common reasons for ED consumption was pleasure or “to have a good time” [34],[36]. The least cited reason was “to keep active” (7.2%), which, if we consider the athletic origins of ED [37], could be a paradox. In fact, several studies have correlated ED consumption with a sedentary lifestyle, revealing that hours of television watching are significantly associated with ED consumption [38],[39].

The most reported ED consumption frequency by the total sample was 1–3 times per week, which could be related to weekend consumption and nightlife. According to a recent study, young people who reported a more active nightlife or those who returned home later constituted a particularly high-risk group for ED consumption (prevalence of consumption > 60%) [11]. This leads us to consider ED consumption with alcohol. In our study, 15.5% of the participants consumed ED with alcohol, and this percentage is lower than that reported by other authors such as De Giorgi et al., who calculated a value of 37.0% [20]. If we consider the youngest participants as reference (14–18 years), the percentage is closer (32.1%). Hence, they constitute the group with the highest percentage by age (*p* < 0.001). Alcohol consumption was not quantitatively determined in our study. Nevertheless, this correlation is widely known, as some studies have pointed out that ED consumption is higher among those who report risky alcohol consumption [40] or that alcohol consumption is positively associated with ED consumption [29],[30]. A systematic review recently published on substance use and ED consumption among adolescents affirmed that alcohol is the most extensively researched substance positively linked with ED consumption [40]. The combined intake of alcohol and ED is rooted in their known psychopharmacological interaction, which was also quantitatively assessed in a recent meta-analysis [41]. Consumers who mix alcohol with ED drink significantly more alcohol than those who drink alcohol without ED. In fact, as previously reported, the depressant effect of alcohol is countered by the stimulant effect of ED, resulting in significantly lower sedation [40].

There are other factors positively associated with ED consumption, such as gender [16],[39],[42],[43]; this was also observed in our results, as male participants were three times more likely to consume ED than female participants (*OR* = 2.94, *CI* = 1.76–4.93, *p* < 0.001). However, it is also interesting to note that, in our study, there could be selection bias in the study population due to the use of the online questionnaire, such that the number of male participants is small in comparison to the number of female participants.

Another factor often associated with ED consumption is vaping [25],[26],[44]–[47]. As per a recent systematic review, it has been established that a prevalent trend of consuming ED alongside various substances, such as alcohol and vaping, exists. In two of the longitudinal investigations reviewed, ED use significantly predicted initiation of vape use, although the inverse relationship was not confirmed in a third study [40].

If we look at our overall results, we see that the youngest participants (aged 14–18) most frequently reported consuming ED for pleasure and mixing them with alcohol. However, participants in this age range also acknowledged the harmful effects of these drinks less frequently. This fact seems extremely significant, considering that adolescence is a susceptible stage of life in which young adults develop new skills toward independence but remain vulnerable owing to their lack of life experience [48]. Food and beverage companies are recognized for capitalizing on the social vulnerabilities of young adults through image-based marketing tactics for their products. These tactics involve utilizing peer ambassadors and celebrity endorsements aimed at promoting an illusion of health, beauty, and success [49]–[51].

An aspect of great importance in this context is the concept of self-objectification, which refers to the tendency to primarily value oneself as a physical object, often sexualized [52]. According to a study conducted with a large sample of adolescents of both genders [43], it was found that this self-perception as a sexual object could be related to the adoption of unhealthy habits such as the consumption of EDs, excessive Internet use, a sedentary lifestyle, or alcohol consumption.

### 4.1. Limitations and strengths

This study has several limitations. First, data collection for this study took place during March and April 2020, amid the COVID-19 pandemic, so the total lockdown experienced during those months may have influenced participants' responses. Similarly, this exceptional situation in which the entire Spanish population found itself during those months hindered the dissemination of the questionnaire and influenced the sampling technique used (non-random sampling). This circumstance may have introduced bias in participant selection. Second, the online questionnaire format could have caused response bias due to acquiescence, even though the questions were simplified to shorten completion time. However, it is important to note that Eckman et al. (2006) found that bias from online questionnaires may not be significantly greater than that from paper surveys [53]. Therefore, the generalizability of our results is limited due to the non-probability sampling and the aforementioned biases. Additionally, as this was a voluntary survey, it is possible that participants who were more aware of the issue were more likely to participate. There is also a limitation regarding the assessment of alcohol consumption, as our study did not analyze variables such as the type of alcoholic beverage (low *vs*. high alcohol content/volume) mixed with ED. However, this could constitute an interesting area of research aimed at exploring potential associations between ED consumption and alcohol consumption. Despite these limitations, while this work cannot be considered representative of the entire Spanish population, it achieved a wide reach and can serve as a valuable starting point for future, more specific studies, such as the consequences of ED consumption in the adolescent population in their biopsychosocial health. On the other hand, one of the strengths of this study lies in the fact that the questionnaire was developed based on reviewed literature and was consensus-driven with a panel of experts using the Delphi method. Furthermore, another potential strength of the study is the large sample size utilized (387 participants).

## 5. Conclusions

Regarding the consumption habits of ED, our study results indicate that the youngest participants in our sample are the most inclined to mix ED with alcohol. Moreover, they are also the ones who least frequently recognize the dangers derived from its consumption. EDs pose health risks. Globally, Nadeem et al. [1] observed frequent adverse effects such as insomnia (35.4%), stress (35.4%), and depressive mood (23.1%). On the other hand, the Scientific Committee of the Spanish Agency for Food Safety and Nutrition (AESAN) recommends that ED be marketed in containers with volumes not exceeding 250 mL, instead of the usual 500 mL, in order to minimize exposure to psychoactive and stimulant substances harmful to health such as caffeine and taurine [54]. Despite the evidence warning about the health risks of ED consumption, EDs are very popular among young people and adolescents, which could be associated with the viral marketing used by brands.

### 5.1. Public health policy

It seems extremely important to include not only policy measures regarding public health but also psychoeducation on self-objectification. Therefore, social and healthcare policies should be proposed with the objective of reducing ED consumption, especially among the younger population, since it is associated with other risky behaviors and the occurrence of adverse events.

## Use of AI tools declaration

The authors declare they have not used Artificial Intelligence (AI) tools in the creation of this article.
